# Round the Hospitals

**Published:** 1918-03-02

**Authors:** 


### ROUND THE HOSPITALS.

OUR Foreword this week, "Supporters of the College," sufficiently demonstrates the fact that the College of Nursing is the voice of the leading matrons now responsible for the future of nurses and nursing. But those who sit on the Council are but representatives of the very numerous body of influential women holding like posts of authority and influence who have already joined the College and undertaken to serve its interests by example and precept. The number of nurses turned out annually in the training-schools, we have enumerated, would alone suffice to ensure such a constant accession of new supporters to the College that its future may be considered assured. But it is not merely the newly trained who come under the influence of these heads of the Nursing Service in the country. Their example tells in far-distant regions, among nurses serving in a hundred different capacities, who look for the lead of their former matron before taking the word of anyone else on matters affecting

☛ Previous articles appeared December 8, 15, 22, January 5, 12, 26, and February 2.

**ROUND THE HOSPITALS—(continued).**

nursing. The matrons of the future, too, are now in process of being formed in all these establishments where the College has been hailed with hope and fervour on the strength of its credentials. For many years past the nursing world has awaited the lead of the matrons whose names we have given. Now the moment has come, and they speak with no uncertain sound. The long list of training-schools in every part of the country, published from time to time, from which new members of the College have been pouring in, testifies to the response nurses are making to the call.

That all these recognised leaders of nursing progress and opinion have given their verdict in favour of the establishment of the College of Nursing is much, and very much. Their pioneer work in connection with it has meant to each and all of them a serious sacrifice. One and all they are very far from intending to dominate or "run" the College now it is in being. On the contrary, they have thought out an admirable scheme through which, at the first election in April next and at every subsequent yearly election, one-third of the places on the Council are to be filled by election on the nomination of members of the College, and they anticipate that in this way the services of representative nurses who do not hold any particular office of authority will be secured. It is the declared wish of all the matrons on the Council that once the foundations have been securely laid in the interests of all nurses the members shall take over for themselves the responsibility of deciding by whom the College affairs shall be administered. Thus, while we lay stress on the boundless influence secured by the presence on the Council of the heads of the leading training-schools in the kingdom, we would like to emphasise the disinterested character in which from the outset this influence has been used. Not one of these women, to whom the College owes so much, has dreamt of attempting to secure for herself permanence in office. The only aim of all has been to facilitate the expression of free choice, eliminate all possibility of intrigue, and set the College on its feet as a self-governing democratic institution.

The meeting of the College of Nursing at Liverpool, which we report fully on pages 467 to 471, was wonderfully successful in drawing large numbers (the Council Chamber was quite full, with all space filled with extra chairs), and in the spirited character of the speeches, which held the audience from start to finish. Those present could be felt to be sympathetic and intensely interested. One of the events of the gathering was the able speech delivered by the matron of the Royal Infirmary, Miss Cummins. At the close of the proceedings she received an agreeable surprise in the shape of a donation of £100 from the father of one of her own nurses, just completing her training, who was inspired by what he heard at this meeting to offer this substantial token of sympathy with the College aims. Another £100 was contributed by a member of the honorary staff of the Liverpool Royal Infirmary, who also was moved by the occasion to become a cheerful giver.

A meeting of members of the College of Nursing was held at the Royal Infirmary, Manchester, on Wednesday, February 20. There were 110 nurses present. The question of forming a local Centre was discussed, and it was decided to start one at once. Miss Smith, R.R.C., was elected as local representative, with Miss Pilgrim and Miss Earl as hon. secretaries. It was decided, as far as possible, to hold meetings monthly and to try to develop the Centre, both on the educational and social side. The coming opportunity of voting for members for the Council was also thoroughly discussed; the members fully realise their privilege and responsibility in this matter.

Miss Thorold, the matron for thirty-eight years of the Middlesex Hospital, passed away a short time back almost as though entering a gateway before which she had been waiting in happy expectancy. Few women whose lives have been spent in nursing had so many vivid outside interests. An omnivorous reader, an indefatigable traveller, a discriminating collector, her knowledge abounded in many uncommon directions. But her own hospital was the dominating interest of her life. Her reign extended almost over the entire period during which trained nursing developed, and Miss Thorold took a very active part in helping to secure for nurses adequate pay and good surroundings. Her Nurses' Home, when she got it built, was perfect in every particular. It was part of the Middlesex system to attach the nurses and sisters to the continued service of the hospital by generous treatment and liberal pensions. The result was a strong sense of *esprit de corps* and a fine spirit of humanity, which considered not merely the patient's body, but also his imagination and soul. Miss Thorold's own rule was to visit every ward herself twice in the course of the twenty-four hours, and by long habit and natural gift combined she came to know every patient by name and sight out of some 350, was intimately acquainted with their condition, and was generally possessed of curiously exact details about their friends and circumstances. In the matter of physique Miss Thorold was active and tireless to a truly remarkable extent. Very few hours of sleep did she ever allow herself—usually but four or five—and much was accomplished in the midnight and early morning hours, which found her still reading or writing or making her last round. She was a faithful and staunch supporter of the Royal British Nurses' Association. She was never known to break an engagement, and could at all times be depended on as a most loyal friend and wise counsellor. The successive improvements and extensions which have placed the Middlesex Hospital in the van of progress were, always, welcomed and promoted by Miss Thorold in full faith in the promise of the

**ROUND THE HOSPITALS—*(continued)*.**

future, a promise she watched in course of fulfilment during her long term of office. In her exquisite lace-edged cap and with the air of benign authority which sat so well on her she was a striking personality in the institution. Doubtless a memorial worthy of her will be placed in the chapel, unique in its plan and decoration, which she loved so well, where the names of many who have served the hospital with entire devotion are already inscribed.

The question of industrial fatigue, now being investigated in many different quarters, is one which bears directly on that of nurses' hours on duty. Lord Henry Bentinck gives an interesting account in the current number of the *Contemporary Review* of experiments and tests carried out in regard to the relation between hours of labour and output. Briefly, so far all goes to show that shorter hours favour increased output, as well as increased efficiency. The actual quantity of work accomplished has been found to be considerably more in a forty-eight-hour week than in a fifty-four- or sixty-hour week. Men and women engaged on piecework find they can earn more in proportion to a reduction in the time they devote to it. For instance, women who worked twelve hours a day at making surgical dressings could not compete as regards speed and total quantity turned out with those who put in only eight hours a day, the average output rising as much as fourteen bobbins an hour as soon as the hours of labour were lessened. In every occupation there is an optimum output at a certain length of day. When this time is overstepped a decrease takes place in production.

The bearing of this on the nurses' hours on duty is obvious. True, nurses' labours cannot be measured by bulk, and a poor day's work from a tired nurse is not so easily detected in the ward as in the factory. But admittedly nurses who are at the top of their form, well rested, with minds capable of intense concentration on the matter in hand, are worth twice as much as the harassed and overburdened. Always in an understaffed institution, where the principle of "drive" is in force, there is loss from the inevitable slackening or breakdown from time to time of the human machine working beyond its normal capacity. Always there is the fear lest some vital omission may be the result of tired nerves. Always there is a deterioration in the tone of the wards, and the patients suffer—usually silently. It is of great importance to the nursing profession that the "optimum" should be ascertained. Will matrons who have experimented in this direction favour us with their views on the number of hours a week which nurses and sisters can put in to the best advantage for the institution—that is to say, in complete possession of their best powers?

We hear a very good account financially of the Imperial Nurses' Club in Ebury Street. The good management which made it a success from the outset continues. The catering is so well done that members' payments cover current expenses, a feat which all experienced housekeepers must admire. The accounts are kept by a qualified accountant, and have always shown a credit balance. For this reason there should be a prompt response to the appeal for £500 to clear off initial expenses, and for the additional £500 needed to furnish the adjoining house and give more sleeping accommodation. Both the lady superintendent, Miss Mayers, and the assistant superintendent, hold honorary appointments, and there is a strong and businesslike committee.

It is not long since an aggrieved V.A.D. helper brought an action in the King's Bench Division against the matron of an auxiliary hospital for daring to report at headquarters that the lady in question "was unsuitable for hospital work." The verdict was given in favour of the matron, and Mr. Justice Darling said the action ought never to have been brought. Now we learn that the matron of the Cottage Hospital at Sutton Coldfield, who discharged one of the district nurses at the hospital in January 1915, has summoned her late employee "for writing and publishing false and defamatory libels" concerning her. The nurse reserved her defence and was committed for trial. We abstain from comment while the case is *sub judice*. But that so much unpleasantness, to say the least of it, should result from the performance of one of the habitual duties of a matron illustrates the fact, not always appreciated among the rank and file, that the matron's duty of pronouncing on a nurse's fitness for her duties demands considerable courage.

Among the souvenirs cherished most reverently by the sisters of war hospitals abroad are the letters they receive from former patients. There are some of these which breathe fresh life into a weary woman, and of this sort is one from an old Regular, quoted in a recent issue of the *Spectator*. "Yes, Sister, I am entitled to the Mons Star. I shall be a trifle proud of it, you can guess, but I am prouder of the fact that it is through your hard work and perseverance that I have still got two legs. If you do not get a Mons Star yourself, you can have mine, if you like. I should be very proud to pass it on to you, because I think you are as much entitled to it as I am." Which are we to be proudest of in these days, our fighting men or our nursing women?

The comparatively new system of setting district nurses to attend cases of measles is being followed with conspicuous success, both in the North and in the South. At the annual meeting of the Leeds and District Nurses' Association it was reported that 846 cases of measles had been nursed with the best results. The Queen's Nurses in Brighton and Shoreham who undertake this department have recently been inspected by Dr. Monckton Copeman, L.G.B., who personally visited some of the cases, and pronounced the nurses to be thoroughly trained and "eminently tactful." It was found that in

**ROUND THE HOSPITALS**—*(continued).*
only half the more serious cases of measles which proved fatal had the services of the Queen's Nurses been utilised, and everything points to the fact that the death-rate for this particular disease, so rarely fatal among the well-to-do, could be lowered to vanishing point were the services of the nurse requisitioned in time.

SIR ARTHUR NEWSHOLME, in addressing the annual meeting of the Brighton and District Nursing Association, reviewed the advance of sanitary science during the last seventy years, and showed how it culminated in the advent of the trained nurse. Seventy years ago, he reminded his hearers, "the value and utility of a nurse were measured largely in accordance with whether she had or had not been protected or 'salted' by a previous attack of typhus." He regretted that sanitary authorities do not sufficiently realise the powers they possess under the Public Health Act of 1875 to provide home nursing for any form of sickness among the poor. He looks forward to a large extension of home nursing under the payment by the Local Government Board of half the cost of the nurse's services. Various types of nurses are needed for the multifarious work which awaits us in the future, and what but the College of Nursing can ensure that adequate training for all on which Sir Arthur Newsholme rightly lays stress?

FEW people in London realise the admirable work which the new women police are doing in the streets. In the agglomeration of broken humanity sucked under night after night for want of guidance and restraint are many who come eventually into infirmary or hospital wards to be refitted, if possible, for a new life or end what has proved a tragic failure. To nurses the truth regarding these waifs and strays of civilisation, often pitifully young, is clear enough. Had half the money and energy needed to repair disaster been spent on intervening to ward off danger before it was too late, a useful future might have been their lot instead of suffering and shame. The women police are as a strong, calm defensive outpost for their sisters, often for their brothers also, entangled in dangers they neither understand nor can altogether avoid. The mere arrival on the scene of a woman constable is often enough. Their trained discrimination between an exhibition of restless vanity and that of vice is seldom at fault, and the quiet rest-rooms which they have at their disposal in central situations have proved the turning-point in many careers destined to all appearance to end in the police-cell.

RED CROSS nurses to the number of 200 were sumptuously entertained at Bristol on February 22 by the Ladies' House and Social Committee of the Colonial Institute, and Miss Jennings' clever play "The Rest Cure," was extremely well performed afterwards for their entertainment. A gracious message received from Queen Alexandra in which she expressed her pleasure "that Bristol is to do honour to the nurses whose splendid work is so deeply appreciated throughout the Empire," was the crowning feature of the occasion.

WE have to express our regret that inadvertently the name of the Duchess of Bedford was substituted for that of Adeline, Duchess of Bedford, on page 421 of our issue of February 16.

☛ IT is our invariable practice to pay for all items of news which we may publish. The minimum payment is 5s. Paragraphs of about twenty lines in length—that is to say, of 150 words or less—are preferred. Contributions should be addressed to The Editor, THE HOSPITAL, 28 and 29 Southampton Street, Strand, London, W.C. 2, and, to be in time for the current week's issue, should reach him by the first post on Mondays.

WE regret that owing to pressure on our space we are obliged to hold over several matters of interest, including a report of a meeting in regard to the College of Nursing, which was held at the Dreadnought Hospital, Greenwich, on Friday, February 22.

For "Ierne" on "Camouflage in Nursing Politics" see p. 462; Matrons' and Sisters' Appointments, p. 472; Tact in Sanatorium Management, p. 472.

